# Milk thistle nano-micelle formulation promotes cell cycle arrest and apoptosis in hepatocellular carcinoma cells through modulating *miR-155-3p* /*SOCS2* /*PHLDA1* signaling axis

**DOI:** 10.1186/s12906-023-04168-5

**Published:** 2023-09-26

**Authors:** Saghar Rahnama, Zahra Moazezi Tehrankhah, Fatemeh Mohajerani, Faezeh Shah Mohammadi, Zahra Yousefi Yeganeh, Farhood Najafi, Sadegh Babashah, Majid Sadeghizadeh

**Affiliations:** 1 Department of Molecular Genetics, Faculty of Biological Sciences, Tarbiat Modares University, Tehran, Iran; 2 Department of Resin and Additives, Institute for Color Science and Technology, Tehran, Iran

**Keywords:** Nano-milk thistle, Noncoding RNA, Cell cycle arrest, Apoptosis

## Abstract

**Background:**

Hepatocellular Carcinoma (HCC) is a prevalent form of liver cancer that causes significant mortality in numerous individuals worldwide. This study compared the effects of milk thistle (MT) and nano-milk thistle (N-MT) on the expression of the genes that participate in apoptosis and cell cycle pathways in Huh-7 and HepG2 cells.

**Methods:**

IC50 values of MT and N-MT were determined using the MTT assay. Huh-7 and HepG2 cell lines (containing mutant and wild-type *TP53* gene, respectively) were incubated with MT and N-MT for 24h and 48h and the impact of MT and N-MT on the proliferation of these cell lines was evaluated through a comparative analysis. Cell cycle and apoptosis were assessed by flow cytometry after 24h and 48h treatment in the cell lines mentioned. Real-time PCR was used to analyze *miR-155-3p*, *PHLDA1*, *SOCS2*, *TP53*, *P21*, *BAX*, and *BCL-2* expression in the cell lines that were being treated.

**Results:**

N-MT reduces cancer cell growth in a time and concentration-dependent manner, which is more toxic compared to MT. Huh-7 was observed to have IC50 values of 2.35 and 1.7 μg/ml at 24h and 48h, and HepG2 was observed to have IC50 values of 3.4 and 2.6 μg/ml at 24 and 48h, respectively. N-MT arrested Huh-7 and HepG2 cells in the Sub-G1 phase and induced apoptosis. N-MT led to a marked reduction in the expression of *miR-155-3p* and *BCL-2* after 24h and 48h treatments. Conversely, *PHLDA1*, *SOCS2*, *BAX*, and *P21* were upregulated in the treated cells compared to untreated cells, which suggests that milk thistle has the potential to regulate these genes. N-MT reduced the expression of *TP53* in Huh-7 cells after mentioned time points, while there was a significant increase in the expression of the *TP53* gene in HepG2 cells. No gene expression changes were observed in MT-treated cells after 24h and 48h.

**Conclusion:**

N-MT can regulate cancer cell death by arresting cell cycle and inducing apoptosis. This occurs through the alteration of apoptotic genes expression. A reduction in the expression of *miR-155-3p* and increase in the expression of SOCS2 and PHLDA1 after N-MT treatment showed the correlation between *miR-155-3p* and *PHLDA1/SOCS2* found in bioinformatics analysis. While N-MT increased *TP53* expression in HepG2, reduced it in Huh-7. The findings indicate that N-MT can function intelligently in cancer cells and can be a helpful complement to cancer treatment.

**Supplementary Information:**

The online version contains supplementary material available at 10.1186/s12906-023-04168-5.

## Background

Liver cancer is the fifth most common type of cancer worldwide and the fourth leading cause of cancer death. It is estimated that the disease will cause over 1 million deaths by 2030. Hepatocellular carcinoma (HCC) is the main usual hepatic malignancy and make up 85% of all cases [1, 2]. Different factors may contribute to the development of HCC, including hepatitis B virus infection, chronic hepatitis C virus, alcohol consumption, dietary, non-alcoholic fatty liver disease, chronic liver disease, and carcinogens that are present in the environment. Surgical resection and chemotherapy are therapeutic approaches for these patients. However, many patients with HCC reach advanced stages and become resistant to chemotherapy due to their poor prognosis and delayed diagnosis. Changes in genetic and epigenetic factors play crucial roles in the development of this disorder. Therefore, it is necessary to explore how abnormal gene expression and molecular mechanisms contribute to this cancer and to find new complementary drugs that are effective without side effects [3, 4].

Over the past two decades, the use of natural products as therapeutic agents has received much attention. These kinds of research have become more scientific and resulted in the discovery of plants such as *Silybum marianum* (also known as milk thistle (MT)), which are effective in treating liver disorders [5–7]. *Silybum marianum*, from the Asteraceae family, is an annual/biennial plant that is naturally inbred in the Mediterranean area and now cultivated worldwide due to its features [8]. It is composed of a mixture of flavonolignans which are commonly called silymarin. The main active component of silymarin is silybin which has powerful antioxidant activity and has demonstrated remarkable anti-cancer efficacy in various cancers such as liver [9–11].

Apoptosis is a type of cell death that is regulated by programmed mechanisms that control different metabolic processes, including homeostasis. The pro-apoptotic protein BAX and anti-apoptotic protein BCL-2 regulate processes in apoptotic pathways. *TP53* and *P21* are involved in cell cycle arrest and tumor suppression. Altered expression of these four genes disrupts cell balance and leading to the development of various types of cancers [12–14]. *PHLDA1* (Pleckstrin homology-like domain family A member 1) have reported as an apoptosis inducer gene in different cells [15–17]. *SOCS2* is another protein coding gene with tumor suppressing effects and mediates apoptosis and inflammation through regulating biological processes in HCC [18]. Researchers have shown that nanoparticle-loaded natural products can alter the dysregulated expression of apoptotic and anti-apoptotic genes in different cancers and be considered as complementary drugs for cancer treatment [19].

Owing to the human genome project and sequencing technology improvements, it is a fact that approximately 75% of the human genome is transcribed into RNA, while only 1–3% is transcribed into protein-coding mRNAs [20–22].When the first small non-coding RNAs, lin-4 and let-7, were discovered in *Caenorhabditis elegans*, the world of ncRNAs began to rapidly expand. Despite lacking protein-coding regions, ncRNAs, as functional molecules, have been shown to be involved in different developmental and physiological processes and their deregulation has been implicated in different diseases, including cancer [23–25]. The major class of small noncoding RNAs is microRNAs (miRNAs), which are 19 to 25 nucleotides long molecules that regulates gene expression by directing the RNA-induced silencing complex (RISC) to miRNA-response elements (MREs), thereby suppressing protein production by either blocking translation or degrading the target mRNA [26, 27]. One single miRNA can target multiple mRNAs and impact the expression of numerous genes. The majority of RNAs carry several MREs and are regulated by different microRNAs [28]. This target multiplicity shows that different RNAs compete for limiting sources of miRNAs, thus acting as competitive endogenous RNAs (ceRNAs). Research has indicated that lncRNAs operate as ceRNAs within a regulatory network, whereby they act as molecular "sponges" for specific target microRNAs in order to modulate the expression of mRNAs. These ceRNAs regulatory networks is broadly involved in different cellular mechanisms of cancer [29, 30]. MiR-155 is a noteworthy member of the known oncoming family and significantly upregulated in different cancers such as liver, colorectal, and oral squamous [31].

In this study, we evaluated the anti-HCC efficacy of MT and N-MT on the proliferation of Huh-7 and HepG2 liver cancer cell lines and compared the effect of N-MT and MT on the expression of *miR-155-3p*, *SOCS2*, *PHLDA1*, *TP53*, *P21*, *BAX*, and *BCL-2* in the hepatocellular carcinoma cells. Moreover, we studied the effect of N-MT on cell cycle arrest and induction of apoptosis in these cell lines. We also suggest a ceRNA network role in leading N-MT-treated HCC cells to apoptosis. Furthermore, we showed that *PHLDA1* and *SOCS2* both have MREs for *miR-155-3p* and assess their expression levels in response to N-MT treatment.

## Methods

### Nano milk thistle preparation

Milk thistle seeds, which consist of flavonolignans silybin, isosilybin, silychristin, isosilychristin, silydianin, and silimonin, were prepared from Mazandaran province, Iran. Nanocarrier was prepared by Dr Sadeghizadeh’s molecular genetics laboratory at Tarbiat Modares University, Iran. 96 g of milk thistle seeds were added into 500 ml of 70% ethanol and stirred for 25 h at 50 degrees Celsius. Then, the milk thistle ethanol extract solution was separated from the sediments by a sieve and the water and ethanol solvent was removed by a rotary device at a temperature of 90 degrees Celsius and under vacuum. The amount of extracted sediment was 13.95 g. This amount of sediment obtained by OA400 Carrier was brought to a volume of 558 ml and stirred at 80 degrees for 1 h. The resulting solution contained 25 mg of total milk thistle extract per ml. The preparation of the OA400 carrier has been previously elucidated in our prior research [32, 33].

### Hepatocellular Carcinoma cell lines, cultures condition and proliferations

HDF Fibroblast cell line as a normal cell, wild-type *TP53* Hepatocellular Carcinoma cell line HepG2 and mutant *TP53* cell line Huh-7 were purchased from Pasteur Institute, Tehran, Iran. These cells were cultured in Dulbecco’s modified Eagle’s medium (DMEM, Gibco, USA), supplemented with 10% fetal bovine serum (FBS, Gibco, USA) and antibiotics including 10, 000 μg/mL of streptomycin and 10,000 U/mL of penicillin (Gibco, USA). In addition, the culture medium of HDF cells was consisting of 1mM L-glutamine. The optimal temperature for cell culture was determined to be 37°C and the concentration of CO2 deemed suitable was 5%.

### Cell viability test via MTT assay

MTT assay was utilized to measure viability of cells after MT and N-MT treatment. The HepG2, Huh-7, and HDF cells were seeded in 96-well plate at the density of 7*103, 5*103 and 5*103 cells per well respectively and incubated overnight to allow them to attach. Huh-7 and HepG2 cells were treated with different concentrations of 0–4.5 µg/ml of MT and N-MT (0–4 µg/ml) and HDF cells only treated with various N-MT concentrations (0–4 µg/ml). 24h and 48h after treatment, MTT was added to each well at a 5 mg/ml final concentration. After 4 h of incubation at 37 °C the culture medium was expelled. Followed by the addition of 200 µl of dimethyl sulfoxide (DMSO) for resolving the formazan product to each well, the absorbance of solubilized formazan was measured using a 96-well plate reader named TECAN (Switzerland) at 570 nm. Inhibitory concentration (IC50) was calculated through GraphPad Prism 8 software. 

### Bioinformatic analysis

GSE67504 was downloaded from the GEO database, which contained Huh-7 cells treated with 25 μM silymarin. GSE101728 microarray data of 7 tumor tissues and tumor-free adjacent tissue of hepatocellular carcinoma patients was retrieved from the GEO database, as well (https://www.ncbi.nlm.nih.gov/gds). Analysis of both datasets was done by the use of GEO2R. Cutoff criteria was *P*-value < 0.05 and |log Fold Change|> 0.5. TargetScan (https://www.targetscan.org), miRDB (https://mirdb.org/), StarBase (http://starbase.sysu.edu.cn/) databases were used to predict the microRNAs target specific mRNAs. Enrichr (https://maayanlab.cloud/Enrichr/) and miRPathDB v2.0 (https://mpd.bioinf.uni-sb.de/) were applied to predict biological pathways these mRNAs participate in. Finding the matching seed sequences of miRNAs was determined by RNAhybrid software (https://bibiserv.cebitec.uni-bielefeld.de/rnahybrid/).

### Total cell RNA extraction and Reverse transcription

Total RNA Huh-7 and HepG2 cells were extracted by TRIzolTM reagent (Invitrogen, USA) before and after treatment with MT and N-MT at concentrations below IC50 for 24h and 48h, according to the manufacturer’s protocol, following the RNase-free DNase (Fermentase, Lithuania) treatment. The concentration of resulted RNAs was measured by Nanodrop (Thermo Fisher Scientific, USA) and agarose gel electrophoresis. Afterward, cDNAs were synthetized from the highly purified RNAs (1 μg) using cDNA synthesis kit (Biotechrabbit, Germany). Due to the shortness of mature miRNAs, poly A polymerase enzyme was used to extend 3ʹ of mature transcripts. In order to synthesize the cDNA, Specific primers were used. Total volume of the synthesis reaction was 20 μl, based on the manufacturer’s protocol.

### Real-Time quantitative reverse transcription-PCR (Real-Time qRT-PCR)

1 μL of cDNA was used as a template along with 0.5 μl of each specific primer, mixed to a 20 μl final volume to perform Real time PCR. Real-time PCR was conducted using the RealQ plus 2X Master Mix Green, High ROXTM (Ampliqon, Denmark) 10 μl of this master mix and 8 μl water was used in each reaction. The sequences of primers are listed in supplementary data (Additional file 1). glyceraldehyde 3-phosphate dehydrogenase (GAPDH) and U6 were used as housekeeping genes. Fold changes were calculated by the use of 2 − ΔΔCt method.

### Cell cycle analysis

Huh-7 and HepG2 were cultured in 6-well plates and treated with the given concentrations of N-MT at 24h and 48h. These cells were then collected, centrifuged at 1,300 rpm in 5 min, washed with PBS and fixed with 70% (v/v) cold ethanol for 24h. 50 μg/mL propidium iodide (Sigma, USA) dissolved in PBS were added to the fixed cells, along with 100 μg/mL RNase A (BioBasic, Canada), and 0.1% Triton X-100 (Sigma, USA) were added to the cells. Cells were incubated at room temperature for 30 min in dark position. DNA content of these cells was assessed by the use of FACS Calibur Flow Cytometer (BD biosciences, USA). Extracted data were analyzed using FlowJo software.

### Apoptosis analysis

To evaluate the rate of cell apoptosis, Annexin V-FITC/PI staining was performed based on manufacturer’s instructions (Roche, Germany). The cells were cultured in 6 well plates and 24h and 48h after N-MT treatment (under IC50 doses are given), cells were harvested and washed with PBS, then, 1X binding buffer (10 mM HEPES/NaOH, PH 7.4, 140 mM NaCl, 2.5 mM CaCl2) was used to resuspend the cells. Finally, these cells were stained with Annexin V-FITC/PI and kept at room temperature for 15 min at dark state. The rate of apoptotic cells was evaluated by the use of FACS Calibur Flow Cytometer (BD biosciences, USA). Extracted data were analyzed using FlowJo software.

### Statistical analysis

All experiments were accomplished in triplicates independently. The results of these experiments were reported based on the means ± standard error means (SEM). *P*-values < 0.05 were considered significant. The student t-test was used to compare two groups with each other and find significant differences between them using GraphPad Prism version 8 software.

## Results

### Cell viability analysis

**Fig. 1:**
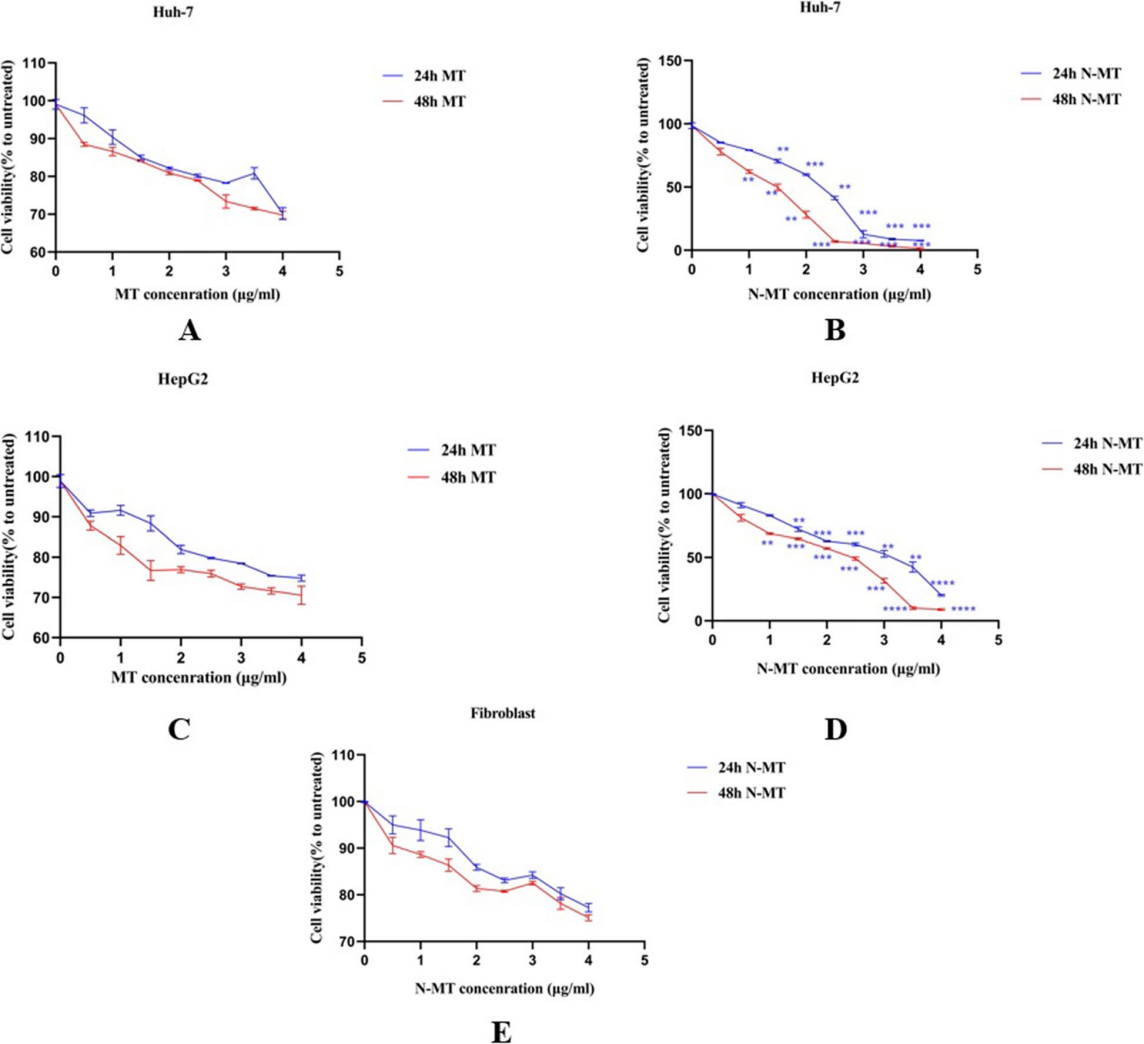
Effect of MT and N-MT on cell viability. **A**, **B** Huh-7 cells were treated with MT and N-MT and their viability was examined by MTT assay after 24h and 48h. **C**, **D** HepG2 cells were treated with MT and N-MT and their viability was examined by MTT assay after 24h and 48h. **E** HDF cells were treated with N-MT and their viability was examined by MTT assay after 24h and 48h. N-MT induces apoptosis in Huh-7 and HepG2 cells in a time- and dose-dependent manner, while the effect of MT extract on the induction of apoptosis in these cells is much lower than that of N-MT. N-MT has no impact on normal cells (fibroblasts) as well. ∗ *P* < 0.05, ∗  ∗ *P* < 0.01, ∗  ∗  ∗ *P* < 0.001, ∗  ∗  ∗  ∗ *P* < 0.0001

MTT assay was conducted to determine the cytotoxic impact of N-MT and MT on Huh-7 and HepG2 cell lines with different concentration of N-MT and MT for 24h and 48h (0–4 µg/ml). Our results showed that N-MT can significantly reduce the viability of Huh-7 and HepG2 cells in a time and dose dependent manner (*p*-value < 0.05). Moreover, MT was not able to decrease the viability of the cells at the under-reviewed times (24h and 48h). To investigate whether N-MT and MT exert toxicity on normal cell lines, HDF cells were treated with different doses of N-MT and MT. The results suggest that N-MT is non-toxic to normal cells and exhibits specific toxicity against the Huh-7 and HepG2 cell lines. (Additional file 2). IC50 values of N-MT in Huh-7 cells was 2.3 and 1.7 μg/ml at 24h and 48h, respectively. In addition, IC50 values of it for HepG2 cells were, 3.4 μg/ml for 24h and 2.6 μg/ml for 48h. Based on these IC50s, we treated Huh-7 cells with 2 μg/ml N-MT for 24h and 1.5 μg/ml N-MT for 48h and HepG2 with 3.1 μg/ml N-MT for 24h and 2.3 μg/ml N-MT for 48h (Fig. 1).

### Bioinformatic results

**Fig. 2:**
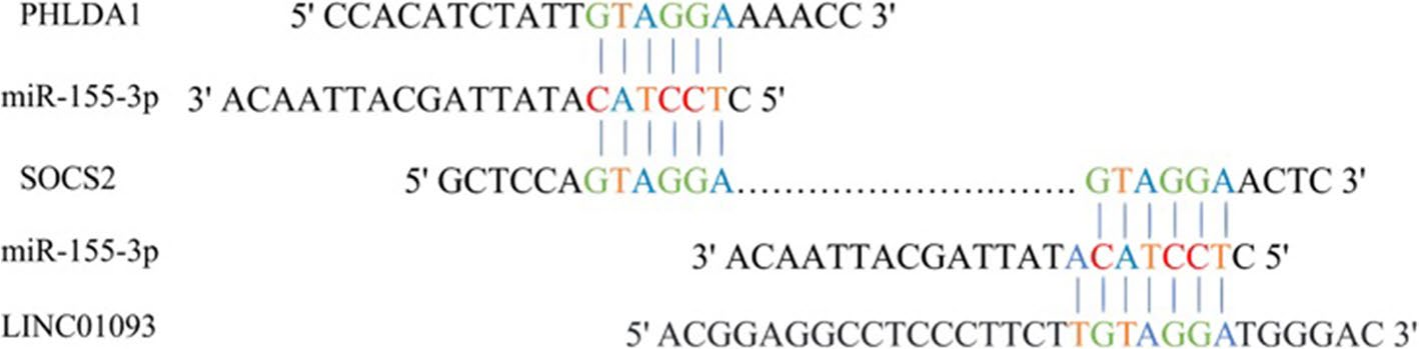
Predicted *LINC01093* and 3’ UTR of *SOCS2* and *PHLDA1* matched with the seed sequence of *miR-155-3p*

We selected PHLDA1 and SOCS2 as candidates according to the bioinformatic analysis of the microarray datasets GSE67504 and GSE101728. Based on analysis of GSE10172 dataset, we selected *LINC01093* (Table 1). Using TargetScan, miRDB, StarBase and miRPathDB V2.0 as our resources, miR*-155-3p* was selected for further studies. Applying RNA-hybrid software, the interaction between *miR-155-3p* and *PHLDA1*, *SOCS2* and *LINC01093* was analyzed (Fig. 2). We projected that *LINC01093* may act as a ceRNA and sponge *miR-155-3p* to alter the expression of *PHLDA1* and *SOCS2*. The results of Enrichr and mirpath showed the participation of *TP53*, *P21*, *BAX*, *BCL-2*, *SOCS2*, *PHLDA1*, and *miR-155-3p* in the apoptosis pathway.

**Table 1:** The differential expression of candidate genes according to the microarray analysis

Accession number	Genes	Log2FC	*P*-value
GSE67504	PHLDA1	0.87997913	0.0112722
	SOCS2	1.03411825	0.0352954
GSE101728	PHLDA1	-1.53245164	3.53E-02
	SOCS2	-1.87970839	8.89E-04
	LINC01093	-4.64290139	3.20E-07

### N-MT reduces the expression of *miR-155-3p *that led to increased expression levels of *SOCS2* and *PHLDA1* in Huh-7 and HepG2 cells

**Fig. 3:**
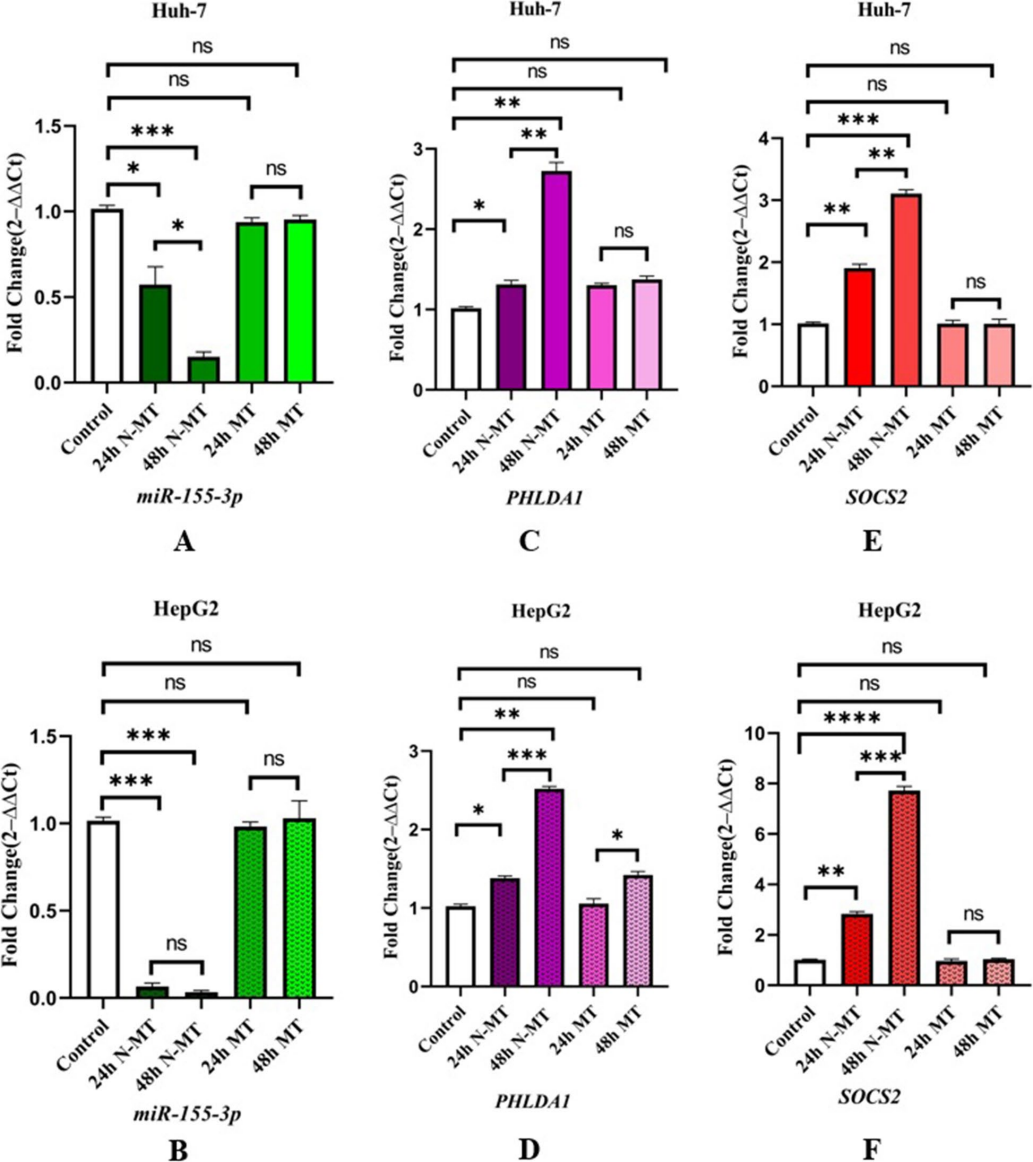
Real-time PCR analysis of three cancer-associated genes after treating the cells with N-MT and MT in Huh-7 and HepG2 cells. Huh-7 cells were treated with 2 and 1.5 µg/mL N-MT and MT for 24h and 48h, respectively. HepG2 cells were treated with 2.3 and 3.1 µg/mL N-MT and MT for 24h and 48h, respectively. **A**,** B** *miR-155-3p*, **C**,** D** *PHLDA1*, **E**,** F** *SOCS2*. The mRNA expression of the cancer-related genes was evaluated by the optimized real-time PCR method at 24h and 48h after treatment. The results are presented as the mean mRNA expression fold changes compared to the untreated. The analyses indicated that N-MT caused *PHLDA1* and *SOCS2* mRNA level upregulation, while *miR-155-3p* gene expression decreased in both cell lines compared to the untreated cells. ∗ *P* < 0.05, ∗  ∗ *P* < 0.01, ∗  ∗  ∗ *P* < 0.001, ∗  ∗  ∗  ∗ *P* < 0.0001, ns: non-significant

Quantitative real-time PCR was performed on MT-treated and N-MT-treated Huh-7 and HepG2 cells, and both untreated cells. The results indicate that N-MT considerably declined the expression level of *miR-155-3p* (Fig. 3A, B) while cause an elevation in the expression level of *PHLDA1* (Fig. 3C, D) and SOCS2 (Fig. 3E, F) compared to the untreated group in both cell lines (*p*-value < 0.05). Our results demonstrated that MT had no significant effects on the expression of *miR-155-3p*, *PHLDA1*, and *SOCS2* in the same concentration of MT in Huh-7 and HepG2 cells after 24h and 48h (Fig. 3).

### N-MT suppresses the expression of mutant *TP53* in Huh-7 cells while increases wild-type form in HepG2 cells

**Fig. 4:**
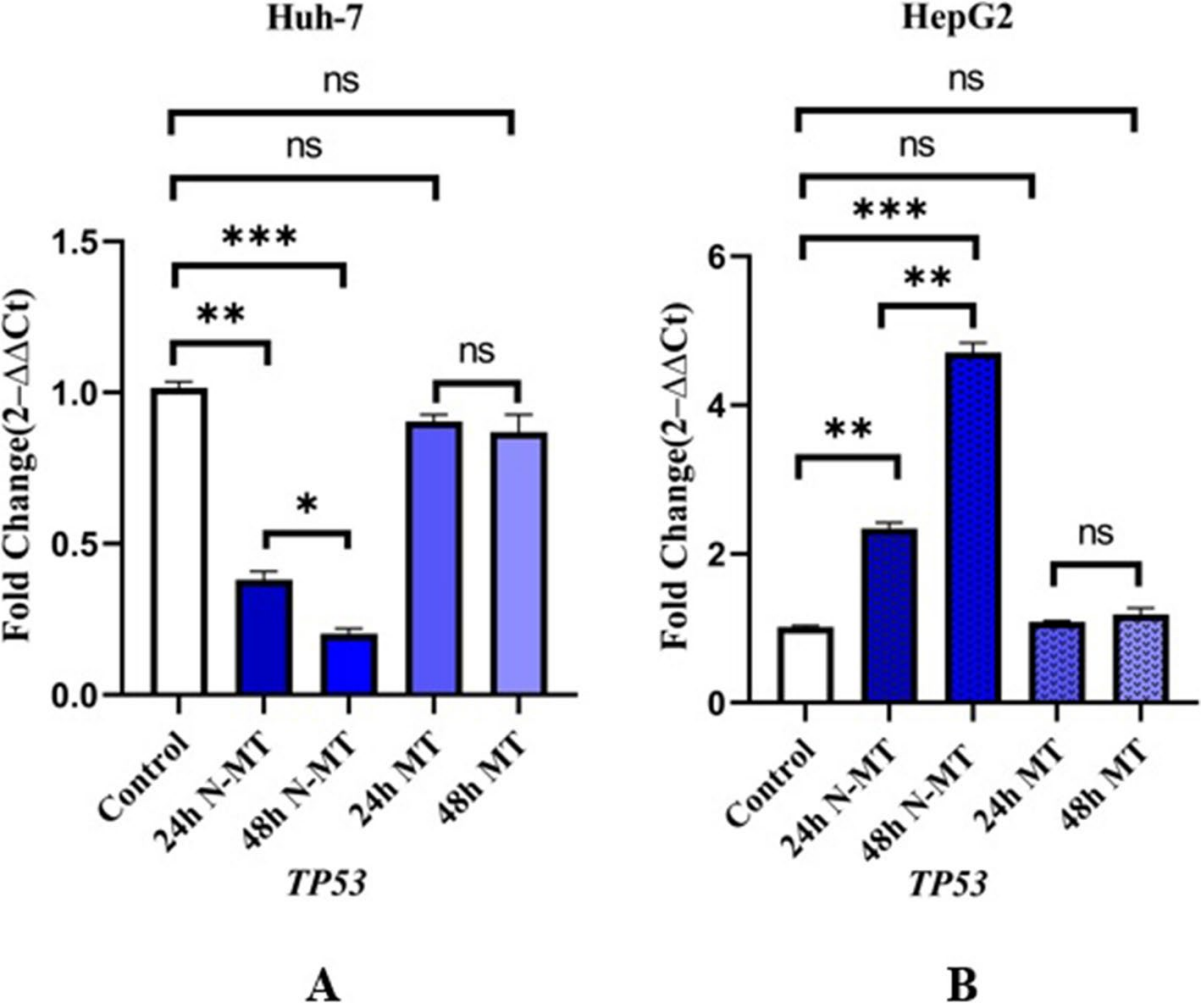
Real-time PCR analysis of the *TP53* gene after treating the cells with N-MT and MT in Huh-7 and HepG2 cells. Huh-7 cells were treated with 2 and 1.5 µg/mL N-MT and MT for 24h and 48h, respectively. HepG2 cells were treated with 2.3 and 3.1 µg/mL N-MT and MT for24h and 48h, respectively. **A** Huh-7, **B** HepG2. The mRNA expression of the cancer-related genes was evaluated by the optimized real-time PCR method at 24h and 48h after treatment. The results are presented as the mean mRNA expression fold changes compared to the untreated cells. ∗ *P* < 0.05, ∗  ∗ *P* < 0.01, ∗  ∗  ∗ *P* < 0.001, ns: non-significant

Based on the mutation of *TP53* in Huh-7 and its wild type expression in HepG2, an analysis was required to assess the impact of N-MT on the transcriptional regulation of *TP53* in both of these cell lines. Derived from the Real Time PCR results, it was elucidated that N-MT cause a decline in the expression of *TP53* in Huh-7 cells (Fig. 4A) along with a corresponding enhancement in HepG2 cells (Fig. 4B) (*p*-value < 0. 01). Conversely, the application of MT did not exhibit any notable impact upon *TP53* expression even with equivalent concentrations. Consequently, N-MT exhibit intelligent functionality with regards to cancer cells (Fig. 4).

### N-MT elevates *P21* and *BAX *expression levels and decreases the expression of *BCL-2* in Huh-7 and HepG2 cells

**Fig. 5:**
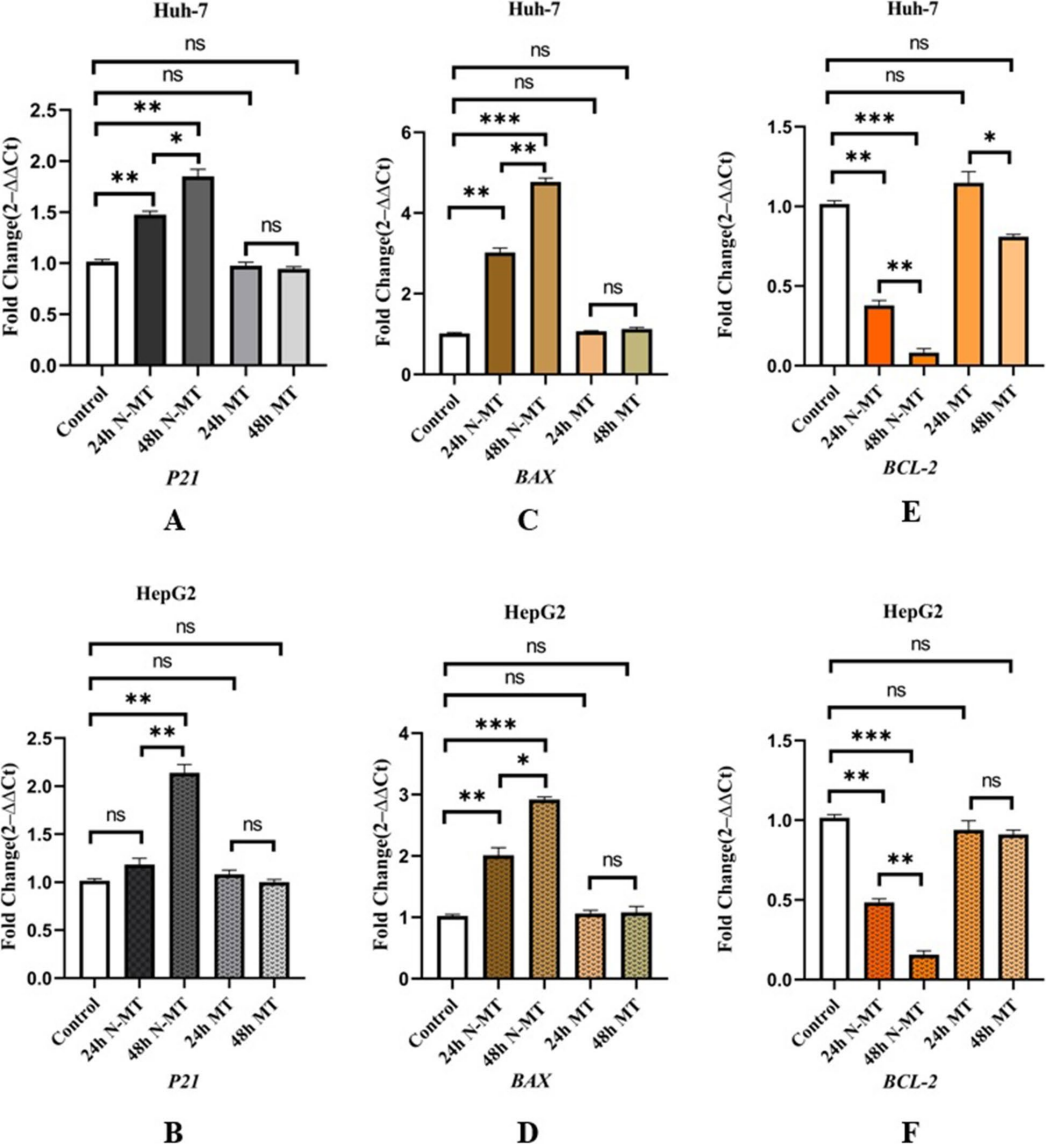
Real-time PCR analysis of three apoptosis-related genes after treating the cells with N-MT and MT in Huh-7 and HepG2 cells. Huh-7 cells were treated with 2 and 1.5 µg/mL N-MT and MT for 24h and 48h, respectively. HepG2 cells were treated with 2.3 and 3.1 µg/mL N-MT and MT for24h and 48h, respectively. **A**,** B** *P21*, (**C**,** D**) *BAX*, (**E**,** F**) *BCL-2*. The mRNA expression of the apoptosis-related genes was evaluated by the optimized real-time PCR method at 24h and 48h after treatment. The results are presented as the mean mRNA expression fold changes compared to the untreated. The analyses indicated that N-MT caused *P21* and *BAX* mRNA level upregulation, while *BCL-2* gene expression significantly decreased in both cell lines compared to the untreated cells. ∗ *P* < 0.05, ∗  ∗ *P* < 0.01, ∗  ∗  ∗ *P* < 0.001, ns: non-significant

Due to the fundamental role of the apoptosis pathways in cancer cells, our study undertook an evaluation of the impact of N-MT and MT on the expression of apoptotic genes. The administration of N-MT resulted in a significant upregulation of *P21* and *BAX* gene expression (Fig. 5 A, B, C, D), as well as suppression of *BCL-2* gene expression in both Huh-7 and HepG2 cell lines (Fig. 5 E, F) (*p*-value < 0. 01). Conversely, the application of MT did not impose any considerable effects on the expression level of these genes in these cell lines when administered at the same concentration of N-MT after 24h and 48h (Fig. 5).

### N-MT arrests cell cycle and promotes apoptosis in Huh-7 and HepG2 cells

**Fig. 6:**
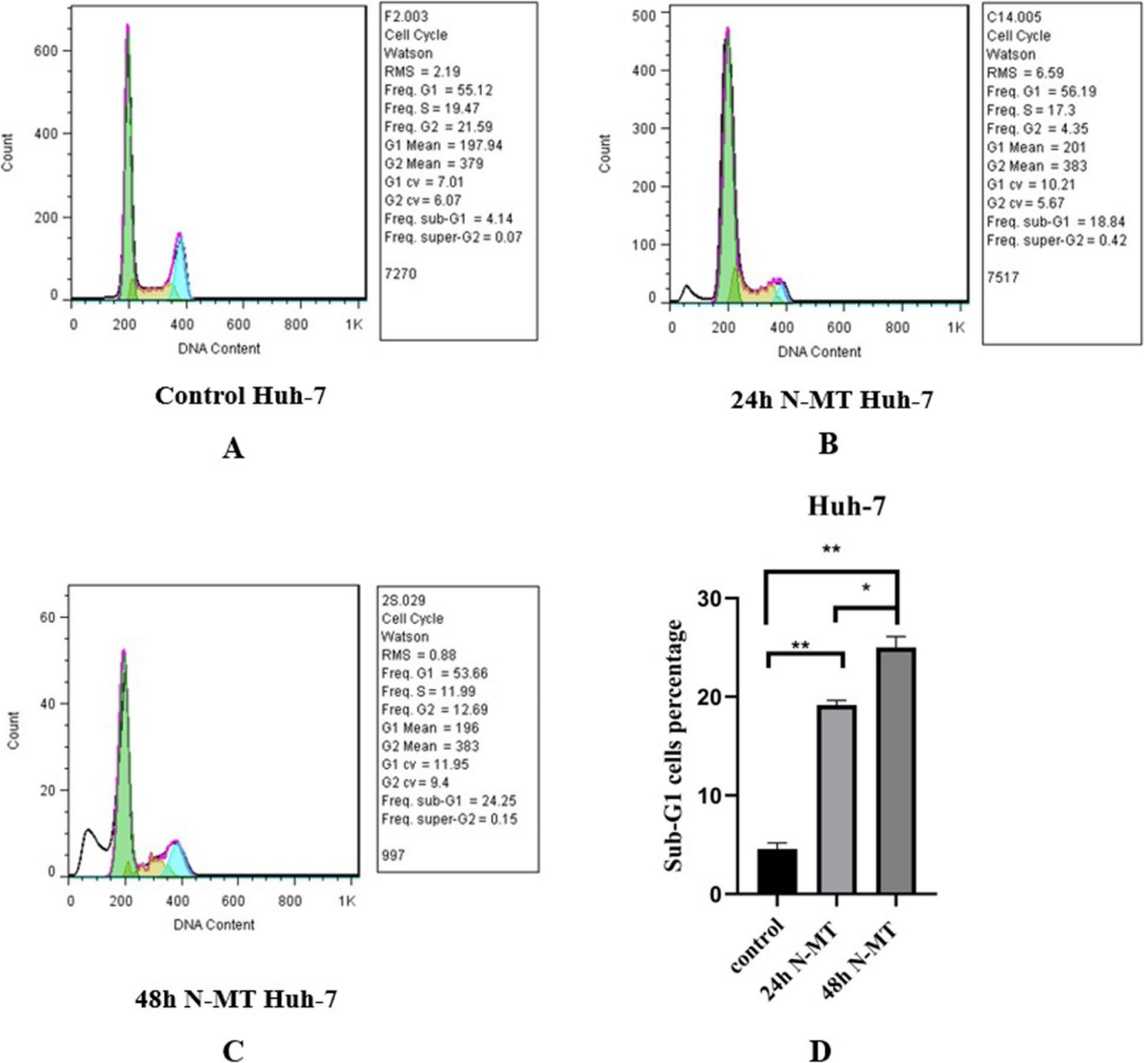
Effect of N-MT on cell cycle in Huh-7 cells, using flow cytometry. Huh-7 cells were treated with 2 and 1.5 µg/mL nano missile encapsulated milk thistle for 24h and 48h, respectively. Panel (**A**) Shows the population of untreated cells in Sub-G1/G1, S and G2/M stages. Panel (**B**,** C**) Show the population of N-MT-treated Huh-7 cells in Sub-G1/G1, S and G2/M stages at two different time points (24h and 48h) after treatment. Panel (**D**) Represents the percentage of the Huh-7 cells in sub-G1/G1 stage after 24h and 48h treatment compared to the control group Sub-G1/G1 cell proportion has increased due to the effect of N-MT in a time-dependent manner. ∗ *P* < 0.05, ∗  ∗ *P* < 0.01

**Fig. 7:**
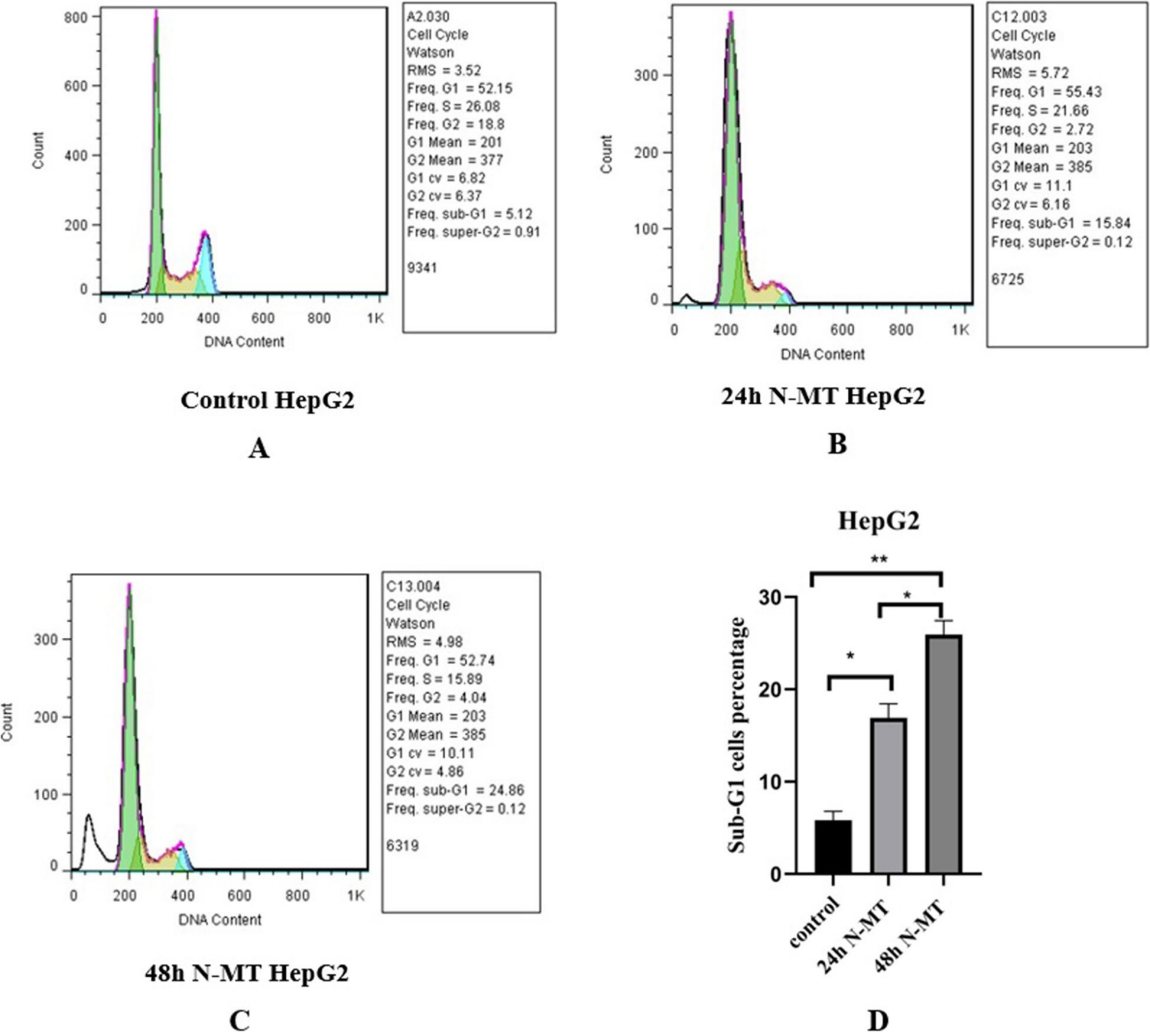
Effect of N-MT on cell cycle in HepG2 cells, using flow cytometry. HepG2 cells were treated with 2.3 and 3.1 µg/mL nano missile encapsulated milk thistle at 24h and 48h, respectively. Panel (**A**) Shows the population of untreated cells in Sub-G1/G1, S and G2/M stages. Panel (**B**, **C**) Show the population of N-MT-treated HepG2 cells in Sub-G1/G1, S and G2/M stages at two different time points (24h and 48h) after treatment. Panel (**D**) Represents the percentage of the HepG2 cells in sub-G1/G1 stage after 24h and 48h treatment compared to the control group. Sub-G1/G1 cell proportion has increased due to the effect of N-MT in a time-dependent manner. ∗ *P* < 0.05, ∗  ∗ *P* < 0.01

**Fig. 8:**
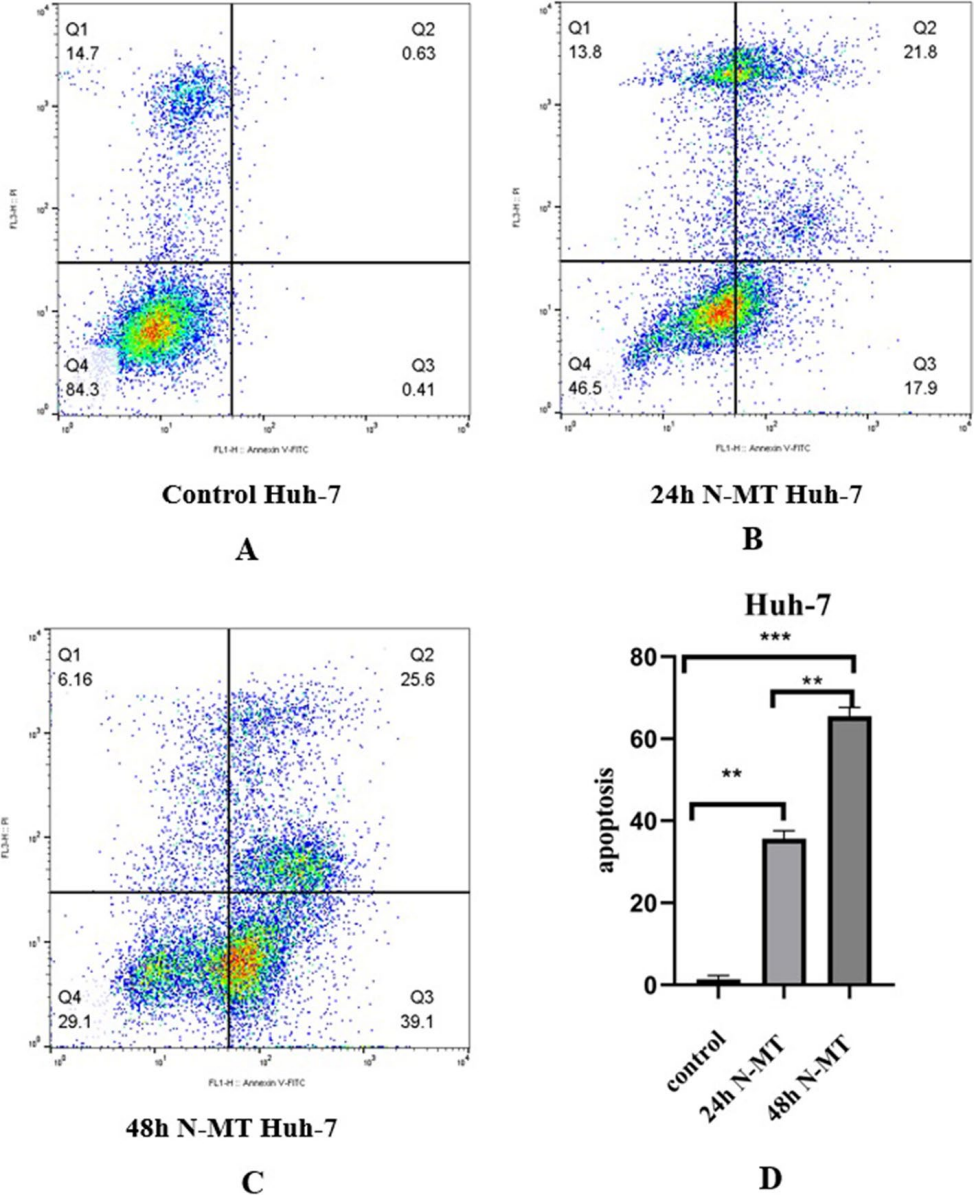
Cellular apoptosis analysis N-MT-treated Huh-7 cells. Huh-7 cells were treated by 2 and 1.5 µg/mL nano missile encapsulated milk thistle, and then, the apoptotic population was measured by the Annexin V-PI assay, using flow cytometry at 24h and 48h. **A** Flow cytometry of the apoptosis for untreated cells,(**B**, **C**) Flow cytometry of the apoptosis for N-MT-treated Huh-7 cells at two different time points (24h and 48h) after treatment, and (**D**) Apoptotic cell percentage of total cells was increased at 24h, and 48h in Huh-7 cell line. ∗  ∗ *P* < 0.01, ∗  ∗  ∗ *P* < 0.001

**Fig. 9:**
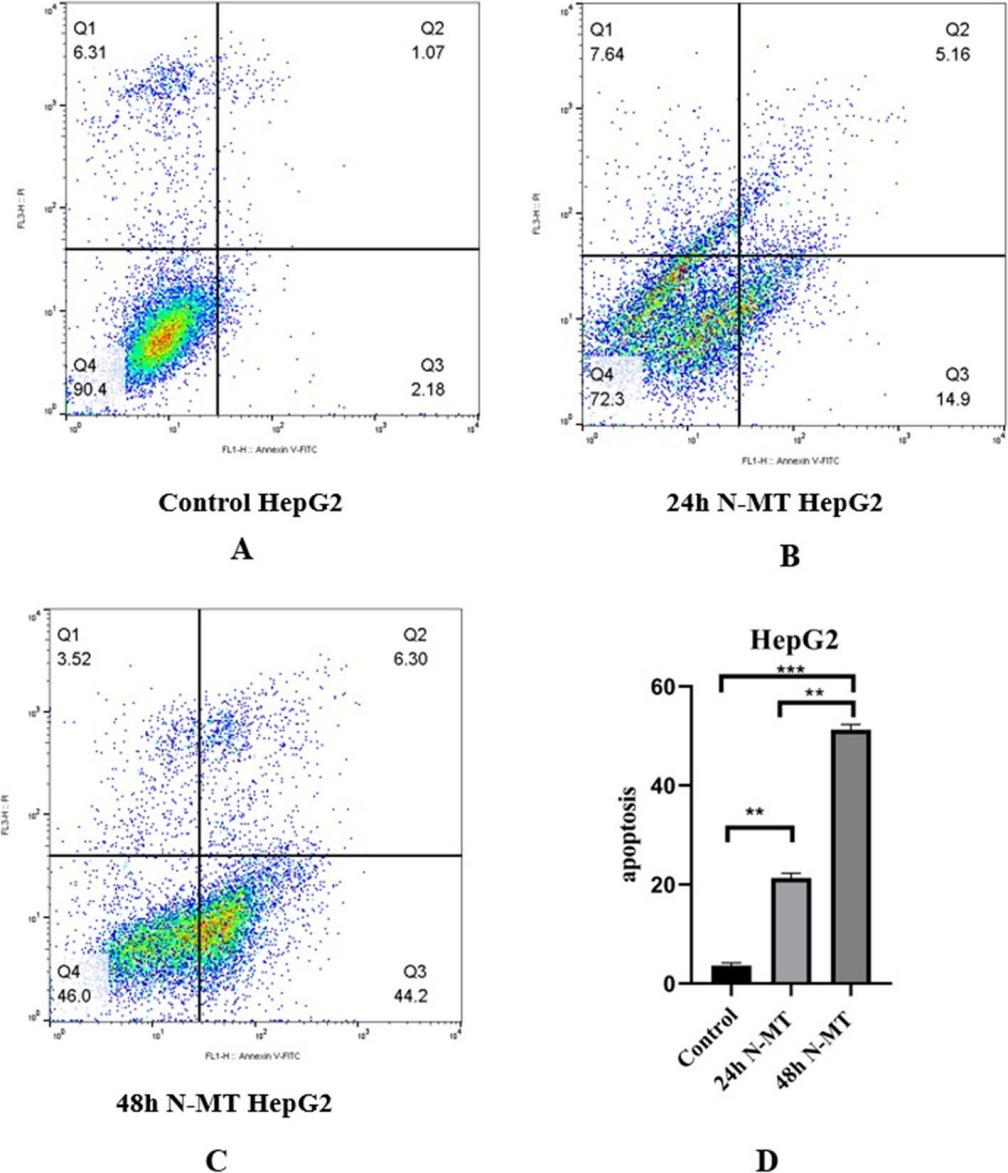
Cellular apoptosis analysis in N-MT-treated HepG2 cells. HepG2 cells were treated by 2.3 and 3.1 µg/mL nano missile encapsulated milk thistle, and then, the apoptotic population was measured by the Annexin V-PI assay, using flow cytometry at 24h and 48h. **A** Flow cytometry of the apoptosis for untreated cells, **B**, **C** Flow cytometry of the apoptosis for N-MT-treated HepG2 cells at two different time points (24h and 48h) after treatment, and **D** Apoptotic cell percentage of total cells was increased at 24h, and 48h in HepG2 cell line. ∗  ∗ *P* < 0.01, ∗  ∗  ∗ *P* < 0.001

Flow cytometry was used to assess the effect of N-MT on the cell cycle arrest and apoptosis in Huh-7 and HepG2 cells. The results of cell cycle analysis showed an increase in the proportion of cells in sub-G1 and G1 stages at 24h and 48h after treatment with N-MT. At 24h and 48h, 18.84% (Fig. 6B) and 24.25% (Fig. 6C) of Huh-7 cells and 15.84% (Fig. 7B) and 24.86% (Fig. 7C) of HepG2 cells were arrested at the sub-G1 phase (p-value < 0.001) (Figs. 6D and 7D) compared to control, respectively (Figs. 6A and 7A). Moreover, PI/Annexin V assay was done and the results of flow cytometry demonstrated the early and late apoptosis in N-MT-treated Huh-7 and HepG2 cells at 24h and 48h. At 24h and 48h, the apoptotic rates in early and late stages of apoptosis were 39.7% (Fig. 8B) and 64.7% (Fig. 8C) in Huh-7 cells and 20.06% (Fig. 9B) and 50.5% (Fig. 9C) in HepG2 cells (*p*-value < 0.001) (Figs. 8D and 9D) compared to control, respectively (Figs. 8A, and 9A).

## Discussion

One of the crucial contributors to the global cancer burden is liver cancer. Its incidence is increasing on a global scale and is anticipated to reach 1 million new cases per year by 2021. Liver cancer consists of a range of malignancies, including cholangiocarcinoma (CCA), hepatocellular carcinoma (HCC), hepatoblastoma (HB), and several other rare tumors like angiosarcoma. Hepatocellular carcinoma (HCC), as the principal histologic type of liver cancer, accounts for more than 90% of liver cancer cases [34–37].

Hepatocellular carcinoma (HCC) is associated with numerous risk factors, including alcohol addiction, age, gender, smoking, exposure to dietary toxins such as aflatoxins and aristolochic acid. However, the most significant causes of HCC are infected with the Hepatitis B virus (HBV) and Hepatitis C virus (HCV) [38, 39].

Similar to other cancers, HCC bears common hallmarks of cancer, including disruptions in signaling pathways that lead to cell proliferation, cell survival, and angiogenesis [40].

The fact that only 5% to 15% of patients suffering from liver cancer are acceptable for surgical resection, the occurrence of drug resistance in patients that apply sorafenib (kinase inhibitor) regimen, and the presence of additional concerns, such as toxicity or drug inefficacy in the long-term application, compel scientists to find better approaches for liver cancer treatment [41].

Due to the fact that natural compounds have low toxicity and side effects, and offer structural similarities between chemical and natural compounds, they seem to be a very suitable alternative to conventional treatments. One of the important points that that must be considered is the therapeutic concentrations of natural compounds in blood and tissue. Obtaining suitable concentrations from experimental results can be very challenging, and their rapid biotransformation in the liver can be important in obtaining the proper curative concentrations of them. The best solution to deliver the proper amount of compounds directly to cancer cells is to transport such agents inside liposomes or dendrimers [42]. Until now, lots of natural compounds have been clinically evaluated against various cancers, and evidences indicated that some of them could inhibit liver cancer development because of their influence on the inflammation, viral infection, oxidant stress, angiogenesis, and metastasis activity [43].

*Silybum marianum L.* is a medicinal plant that has a long history in traditional medicine for treating various human illnesses such as liver disorders, kidney problems, rheumatism, and gastronomic disturbances [44]. Silymarin, a flavonolignan isolated from Silybum marianum, has the potential to reduce the viability, adhesion, and migration of tumor cells by induction of apoptosis and the formation of reactive oxygen species (ROS). In addition, it has antioxidative, anti-lipid peroxidative, antifibrotic, immunomodulating, anti-inflammatory, and even liver regenerating effects [45]. One of the main challenges in applying anti-cancer medicines is the way that the drug will be delivered. The main purposes of different drug delivery systems are designed to improve medication bioavailability, reduce adverse impact, and prevent drug degradation. Novel drug delivery systems have more advantages, including solubility enhancement, bioavailability improvement, less side effects, boosted therapeutic action, stability increase, and better drug distribution [46].

One of the most effective strategies to conquer the unlimited proliferation of cancer cells is promoting cell death. Researchers have shown that the IC50 value for silybin on HepG2 cells was 58.72 ± 2.03 µg/mL and Huh-7 was 17.18 ± 0.97 µg/mL at 24h [11]. In this research, we evaluated the cytotoxic effects of MT and N-MT on hallmarks of cancer, including cell survival and apoptosis in Huh-7 and HepG2 cell lines. Our results illustrate that N-MT promoted apoptosis in Huh-7 and HepG2 cell lines at a much lower concentration than MT, as the IC50 was under 5μg/ml at 24h and 48h.

The Seeds dry extract of *silybum marianum* is named silymarin and mainly includes flavonolignans. The main silymarin flavonolignans are silybin, isosilybin, silychristin, isosilychristin, silydianin, and silimonin which Silybin is quantitatively the major flavonolignan. Silybin is the major bioactive component of the extract and has been confirmed in different studies as a variety of cell-signaling pathways modulator and a potential anticancer agent [8, 47]. In various studies showed, silymarin and silybin have proapoptotic and anti-proliferative effects and can promote G1 arrest in different cancer cell lines. Based on studies, these pro-apoptotic effects are attributed to upregulation of pro-apoptotic genes like *BAX* and *TTP53* as well as *BCL-2* downregulation [48, 49]. Other research also showed that Silymarin also reduced the mitochondrial transmembrane potential of cancer cells, thereby increasing cytosolic cytochrome c levels [50].

The findings of our research indicate that Huh-7 cells treated with N-MT exhibit a significant reduction in *TP53* expression level, whereas HepG2 treated cells demonstrate a substantial increase in the expression of it. This can be consumed those disturbances in *TP53* level of expression after treating HepG2 with N-MT directly effects cytochrome-c release from mitochondria and promotes apoptosis. One of the key factors in cell apoptosis regulation is the cellular *BCL-2/ BAX* ratio; a high ratio makes cells resistant to apoptotic stimuli, while a low ratio induces cell death [51]. Our data indicates that expression of *BAX* and *BCL-2* genes in HepG2 and Huh-7 N-MT treated cell lines compared to untreated cells significantly increase and decrease, respectively. These results suggest that N-MT can induce *TP53* expression in HepG2 cell line and, through this induction, increase the *BAX* expression level and decrease *BCL-2* expression. Reversely, in the Huh-7 cells treated with N-MT, the expression of *TP53* was declined due to the mutation of this gene in this cell line. But while the expression of *BAX* was elevated, the expression of *BCL-2* decreased. It seems that Huh-7 cells treated with N-MT selected another pathway for apoptosis.

*P21* is introduced as a tumor suppressor in the brain and is shown to be as a tumor growth suppression inducer through wild-type *TP53* activity. The most mutated protein in cancers is *TP53*, which is an inducer of *P21* expression in response to cellular stress, such as DNA damage or oxidative stress. Additionally, *P21* has crucial regulatory roles in senescence, apoptosis, DNA damage response, and actin cytoskeleton remodeling*. P21* expression induction due to *TP53* transcription factor activity results in tumor growth arrest through inhibition of cyclin-kinase complex, PCNA, transcription factors, and coactivators [52]. Our results are in line with the mentioned data and reveal that *P21* expression level in N-MT-treated HepG2 and Huh-7 cell lines witnessed a statistically significant increase. This *P21* expression level rise, lead to cyclin-kinases activity inhibition and made a G1 cell cycle arrest based on flow cytometry data.

Bioinformatic analysis anticipates that miRNAs can regulate the expression of more than a third of human protein- coding genes. There are online programs, such as TargetScan, miRDB, StarBase, and Enrichr that can be used to predict potential miRNAs targets and related signaling pathways. Due to our bioinformatic analysis, two targets of *miR-155-3p* named *SOCS2* and *PHLDA1* were selected. *PHLDA1* is known to act as a tumor suppressor by inhibiting AKT signaling pathway and a decreased expression of it has been confirmed in various type of cancers like ovarian and breast [53]. *PHLDA1* is a protein-coding gene expressed in various mammalian tissues and plays key roles in regulating different biological processes in cancer, including cell cycle progression, proliferation, and tumorigenesis. In co-expression network analysis research, *PHLDA1* was shown to be one of the top 10 genes in HCC associated with malignant progression and the prognosis of patients [15, 17]. *SOCS2* is another protein-coding gene which knockdown of it promoted HCC cells proliferation and metastasis and was associated with HCC progression. Acting as a tumor suppressor in hepatocellular carcinoma [18]. 

Our data showed that expression of *miR-155-3p* in N-MT-treated HepG2 and Huh-7 cell lines, is significantly decreased and this may lead to an increase in the expression of its target genes. According to other studies, the role of slybin in decreasing miRNA-155 expression level and the effect of this deduction on the decrease of cell proliferation and the increase of apoptosis in the MCF-7 cell line have been elucidated [54]. Our investigations confirm this information, and our reports showed an increase in the expression of *SOCS2* and *PHLDA1* in both N-MT-treated cell lines. It can be suggested that N-MT may play a role in decreasing cell proliferation through the regulation of these two genes and this miRNA.

**Fig. 10:**
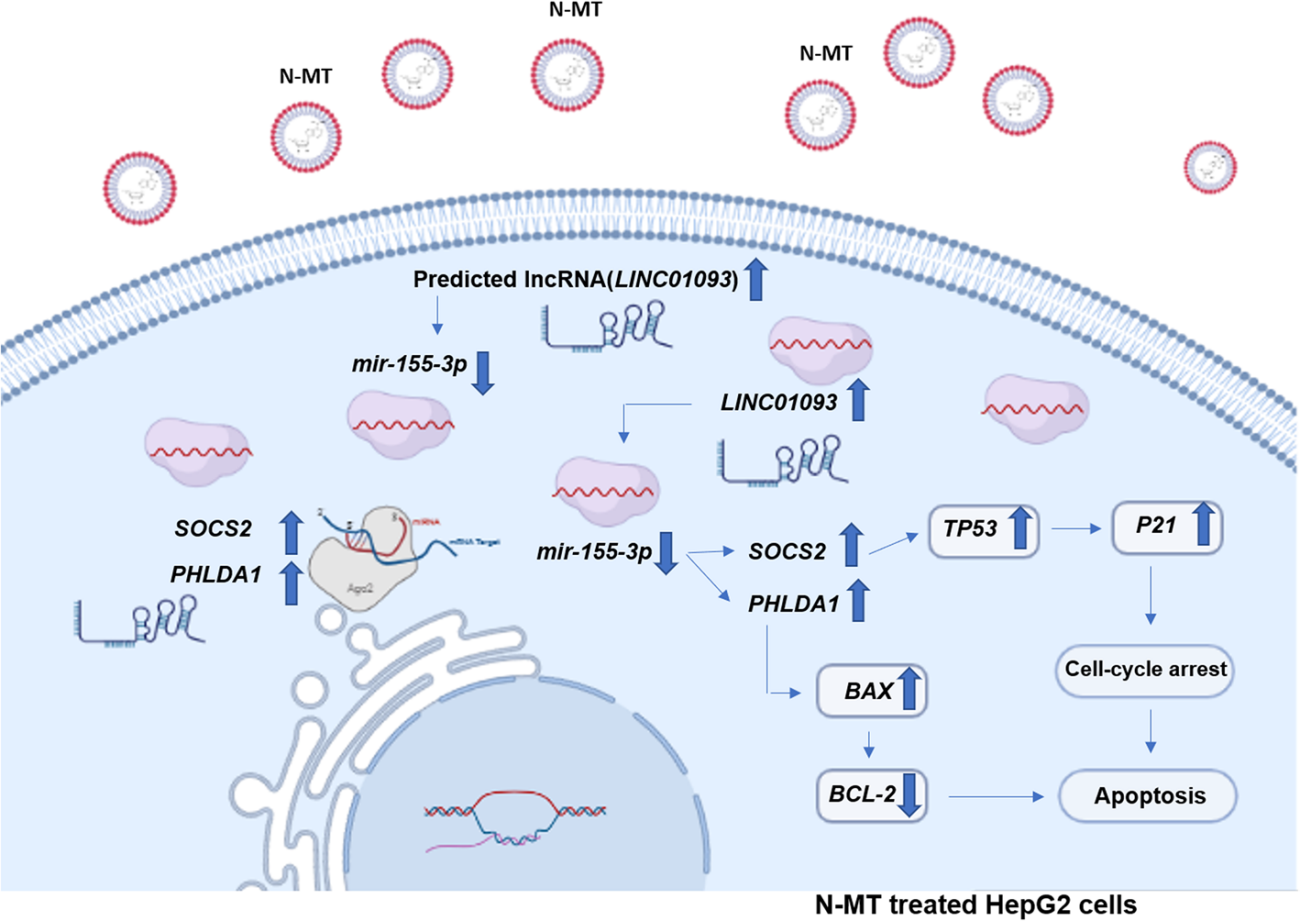
A schematic representation of the predicted ceRNA pathway in a cancer cell treated with N-MT. As can be seen, N-MT-treated cancer cells witnessed an alteration in various genes expression that eventually leads to apoptosis of cancer cells. *LINC01093* has been predicted to have an association with *miR-155-3p* which is decreased and result in the *SOCS2* and *PHLDA1* increment. (This figure was drawn by the BioRender site (https://app.biorender.com)

From our data, a decrease in the expression of *miR-155-3p* in N-MT-treated cell lines can be caused by an increase in unknown lncRNA expression and eventually lead to an increase in the expression of *SOCS2* and *PHLDA1*. These two genes, along with *TP53* and *P21*, have been found to contribute to the apoptosis pathway. Upregulation of these genes results in cell cycle arrest in HCC cells. Additionally, an increase in *BAX* expression levels and a decrease in *BCL-2* expression levels triggers the apoptotic pathway (Fig. 10). We suggested that *LINC01093* may act as a ceRNA and sponges *miR-155-3p* to increase the expression of *SOCS2* and *PHLDA1*. This bioinformatic result needed to be confirmed in laboratory.

## Conclusion

In conclusion, we evaluated the impact of N-MT on the cell cycle arrest and apoptosis of Huh-7 and HepG2 cell lines. The given data showed that N-MT increases the rate of apoptosis in HCC cells and also caused arresting treated cells in sub-G1 cellular phase. Our findings illustrated that utilizing N-MT results in a significant decrease in the expression of *miR-155-3p* which is followed by an increase in the expression of *PHLDA1* and *SOCS2*, compared to MT. This data corroborates our bioinformatics analysis and showed that our nano-herbal drug has a significant potential to apply as a complementary medicine for the treatment of HCC patients.

### Supplementary Information

**Additional file 1:** **Supplementary Table 1. **Primer sequences utilized for real-time PCR.

**Additional file 2:** **Supplementary Figure 1.** Treatment of Huh-7 and HepG2 cells with different concentration of milk thistle loaded in nano carrier and milk thistle extract. A) Huh-7 control B) Huh-7 treatment with N-MT for 24h C) Huh-7 treatment with N-MT for 48h D) Huh-7 treatment with MT for 24h E) Huh-7 treatment with MT for 48h F) HepG2 control G) HepG2 treatment with N-MT for 24h H) HepG2 treatment with N-MT for 48h I) HepG2 treatment with MT for 24h J) HepG2 treatment with MT for 48h. N-MT gave rise to apoptosis of HepG2 and Huh-7 cell, while MT did not have significant effect on the apoptosis of these cells at the same concentrations.

## Data Availability

The datasets used and analyzed during the current study are available from the corresponding author on reasonable request.
