# Supplementary material for: Milk thistle nano-micelle formulation promotes cell cycle arrest and apoptosis in hepatocellular carcinoma cells through modulating miR-155-3p /SOCS2 /PHLDA1 signaling axis
Source: BMC Complement Med Ther. 2023 Sep 26;23:337. doi: 10.1186/s12906-023-04168-5 (PMC10521506; doi:10.1186/s12906-023-04168-5)
Supplement: Supplementary file 1 — Additional file 1: Supplementary Table 1. Primer sequences utilized for real-time PCR. [file 12906_2023_4168_MOESM1_ESM.docx]

| Size of PCR Product (bp) | Chromosomal Location | Primer (5^՜^ 3՜) |  | Gene |
| --- | --- | --- | --- | --- |
| 141 | 12q21.2 | CACCAAATACCGCACCCACA | F | PHLDA1 |
|  |  | TGCTCGTCCCACTTCCTCAA | R |  |
| 187 | 12q22 | TCGGTCAGACAGGATGGTACT | F | SOCS2 |
|  |  | CTGAATTTTCCGTCTTGGTATTCG | R |  |
| 265 | 17p13.1 | TCCTCAGCATCTTATCCGAGTG | F | P53 |
|  |  | AGGACAGGCACAAACACGCACC | R |  |
| 151 | 6p21.2 | ACTCTCAGGGGTCGAAAACGG | F | P21 |
|  |  | GATGTAGAGCGGGCCTTTGA | R |  |
| 183 | 19q13.33 | GCAAACTGGTGCTCAAGG | F | BAX |
|  |  | CAGCCACAAAGATGGTCA | R |  |
| 238 | 18q21.33 | TGGGATGCCTTTCGTGGAACTGTACG | F | Bcl2 |
|  |  | GCCTGCAGCTTTGTTTCATGGTACA | R |  |
| 123 | 12p13 | GTGAACCATGAGAAGTATGACAAC | F | GAPDH |
|  |  | CATGAGTCCTTCCACGATACC | R |  |
| 81 | 21q21.3 | CCCGCCTCCTACATATTAGCAT | F | miR-155-3p |
|  |  | GCGTCGACTAGTACAACTCAAG | R |  |
| 85 | 6p21.33 | AGTGATGATGACCCCAGGTAAC | F | U48 |
| GCGTCGACTAGTACAACTCAAGGTTCTTCCAGTCACGACGTTTTTTTTTTTTTTTTTTV | | | Anchored Oligo(dt) | |
