# Supplementary material for: Milk thistle nano-micelle formulation promotes cell cycle arrest and apoptosis in hepatocellular carcinoma cells through modulating miR-155-3p /SOCS2 /PHLDA1 signaling axis
Source: BMC Complement Med Ther. 2023 Sep 26;23:337. doi: 10.1186/s12906-023-04168-5 (PMC10521506; doi:10.1186/s12906-023-04168-5)
Supplement: Supplementary file 2 — Additional file 2: Supplementary Figure 1. Treatment of Huh-7 and HepG2 cells with different concentration of milk thistle loaded in nano carrier and milk thistle extract. A) Huh-7 control B) Huh-7 treatment with N-MT for 24h C) Huh-7 treatment with N-MT for 48h D) Huh-7 treatment with MT for 24h E) Huh-7 treatment with MT for 48h F) HepG2 control G) HepG2 treatment with N-MT for 24h H) HepG2 treatment with N-MT for 48h I) HepG2 treatment with MT for 24h J) HepG2 treatment with MT for 48h. N-MT gave rise to apoptosis of HepG2 and Huh-7 cell, while MT did not have significant effect on the apoptosis of these cells at the same concentrations. [file 12906_2023_4168_MOESM2_ESM.docx]

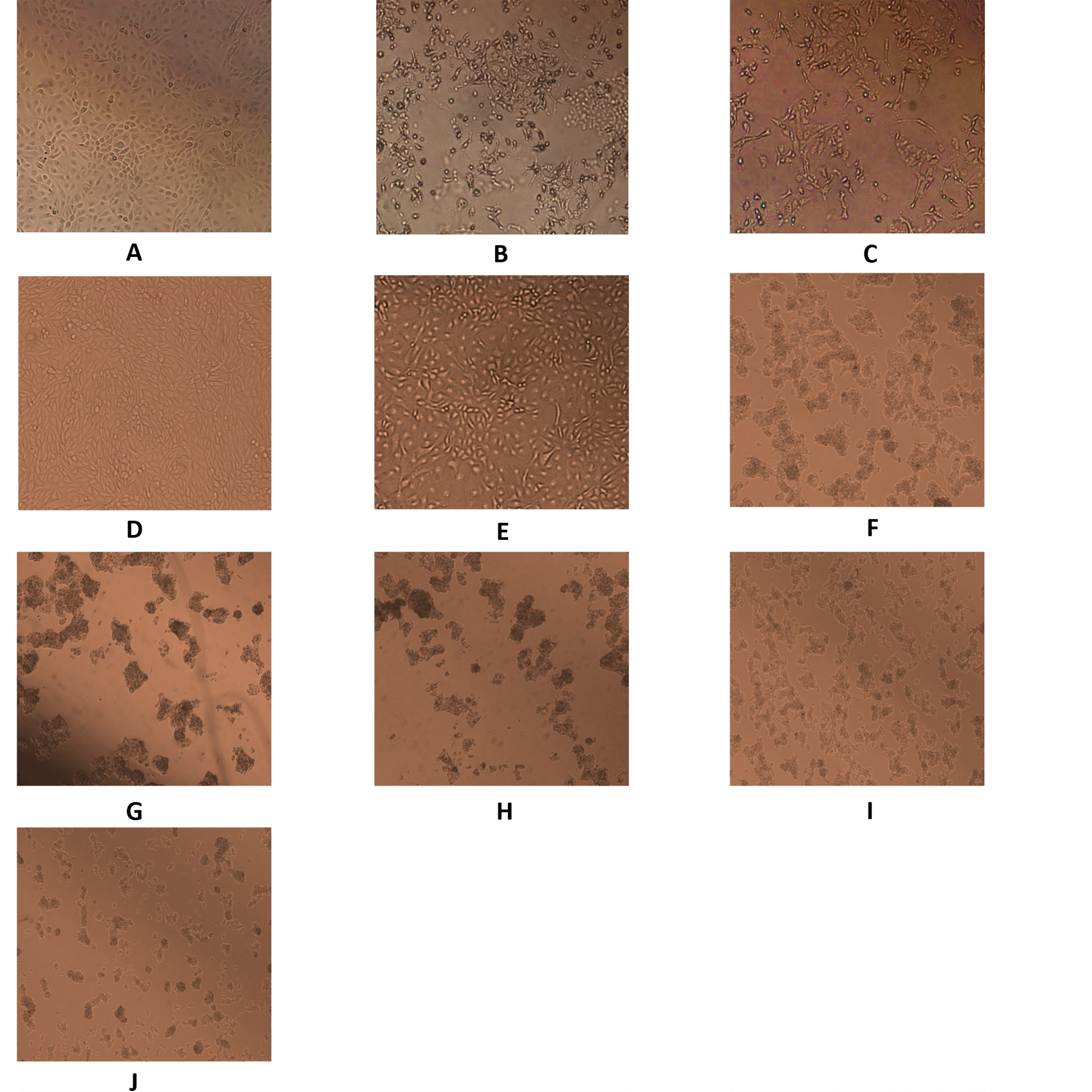


**Supplementary Figure 1.** Treatment of Huh-7 and HepG2 cells with different concentration of milk thistle loaded in nano carrier and milk thistle extract. **A)** Huh-7 control **B)** Huh-7 treatment with N-MT for 24h **C)** Huh-7 treatment with N-MT for 48h **D)** Huh-7 treatment with MT for 24h **E)** Huh-7 treatment with MT for 48h **F)** HepG2 control **G)** HepG2 treatment with N-MT for 24h **H)** HepG2 treatment with N-MT for 48h **I)** HepG2 treatment with MT for 24h **J)** HepG2 treatment with MT for 48h. N-MT gave rise to apoptosis of HepG2 and Huh-7 cell, while MT did not have significant effect on the apoptosis of these cells at the same concentrations.
